# A Scoping Review of the Oral Health Status, Oral Health Behaviours and Interventions to Improve the Oral Health of Children and Young People in Care and Care Leavers

**DOI:** 10.3390/dj12020038

**Published:** 2024-02-09

**Authors:** Joelle Booth, Jo Erwin, Lorna Burns, Nick Axford, Jane Horrell, Hannah Wheat, Robert Witton, Jill Shawe, Janine Doughty, Sarah Kaddour, Skye Boswell, Urshla Devalia, Abigail Nelder, Martha Paisi

**Affiliations:** 1 Centre for Dental Public Health and Primary Care, Queen Mary University of London, Turner Street, London E1 2AD, UK; 2 Peninsula Dental School, University of Plymouth, Drake Circus, Plymouth PL4 8AA, UKlorna.burns@plymouth.ac.uk (L.B.);; 3 Peninsula Medical School, University of Plymouth, Drake Circus, Plymouth PL4 8AA, UK; 4 School of Nursing and Midwifery, University of Plymouth, Drake Circus, Plymouth PL4 8AA, UK; 5 School of Dentistry, Royal Liverpool University Dental Hospital, Pembroke Place, Liverpool L3 5PS, UK; 6 Pathway Oral Health Fellow, Pathway, 250 Euston Road, London NW1 2PG, UK; 7 Patient and Public Involvement Member, Plymouth County Council, Plymouth PL1 3BJ, UK; 8 Royal National ENT and Eastman Dental Hospitals, University College London Hospitals, London NW1 2BU, UK; 9 Peninsula Dental Social Enterprise, Plymouth PL6 8BT, UK

**Keywords:** global oral health, children’s oral health, children looked after, orphans, vulnerable children, unaccompanied refugee asylum minors, foster, oral health status, oral health knowledge, oral health related quality of life

## Abstract

Background: Children and young people (CYP) in care experience poorer physical health and overall wellbeing in comparison to their peers. Despite this, relatively little is known about what their oral health needs and behaviours are. The aim of this scoping review was to provide a global perspective on the oral health status and behaviours of CYP in care and care leavers. It also aimed to synthesise interventions that have been trialled in this population to improve oral health. Methods: Five databases were searched, Ovid Embase, Ovid MEDLINE, CINAHL (EBSCOhost), SocINDEX (EBSCOhost) and Dentistry and Oral Sciences Source (EBSCOhost), alongside grey literature sources up to January 2023. Eligibility criteria were studies that (i) reported on children and adolescents aged 25 years or younger who are currently in formal/informal foster or residential care and care leavers, (ii) pertained to oral health profile, behaviours or oral health promotion interventions (iii) and were published in the English language. Thematic analysis was used to develop the domains for oral health behaviours and interventions. Results: Seventy-one papers were included. Most papers were published from very high or medium Human Development Index countries. CYP in care were found to experience high levels of decay, dental trauma, periodontal disease and poorer oral health-related quality of life. Oral health behaviours included limited oral health self-care behaviours and a lack of oral health-based knowledge. The trialled interventions involved oral health education, supervised brushing and treatment or preventative dental care. Conclusions: This scoping review reveals that CYP in care experience poorer oral health in comparison to their peers. They are also less likely to carry out oral health self-care behaviours. This review highlights a scarcity of interventions to improve the oral health of this population and a paucity of evidence surrounding the oral health needs of care leavers.

## 1. Introduction

The term children and young people (CYP) in care, encompasses a range of scenarios in which children are living away from their biological parents. Terminology to describe the arrangements for CYP in care is fraught with difficulty and makes comparisons across types of care difficult [1,2]. The term ‘foster care’ is used to refer to formal, temporary placements made by order of the state which places CYP in the care of families that have been approved and placements that are supervised [3]. In developing countries, however, the term fostering often refers to kinship care and is more often an informal arrangement that is unregulated by judicial authorities [1,3]. Other examples of alternative care provision include supported child-headed households (where no adult carers are available within the household), community-based care mechanisms and guardianship. Finally, residential care settings are used worldwide and refer to any living arrangement where young people are cared for by paid staff within a designated establishment [1]. Whilst the exact number of children living in care is unknown, it is estimated that around 2.9 million children are living in residential care globally [4]. The highest regional rate of children living in residential care is reported in Eastern Europe and Central Asia at a rate of 585 children per 100,000, in comparison to South Asia and North America, which both have the lowest rate of 77 per 100,000 [4]. 

Evidence suggests that children in care have poorer physical health, mental health and overall wellbeing in comparison to their peers [5,6]. They are also more likely to experience developmental delay and neurodevelopmental disorders [7,8]. Whilst the global number of care leavers is unclear, more than a third of care leavers felt that they left care too early, with feelings of being alone or isolated being common in this group [9]. Care leavers face substantial challenges, and it is estimated that 25% of the homeless population [10] and 24% of the adult prison population in England have previously been in care [11]. Care leavers also face health inequalities with care-experienced individuals being up to four times more likely to suffer poor health in adulthood than those who grew up with their parents [5]. These inequalities transcend to oral health for CYP in care. CYP in care experience higher levels of dental caries, traumatic dental injuries and dental pain than their peers. This is primarily due to the context of their living circumstances, CYP in care can enter the care system with poor health due to poverty, abuse and parental neglect [12]. Maintaining their health once they are in the system is also challenging due to frequent relocation within the care system, variations between placements and the type of care provided. Although CYP in care have higher oral health needs, it has been identified that they can experience greater difficulties in accessing dental services and are less likely to visit the dentist regularly or in line with recall guidance [13,14]. There is a paucity of evidence to suggest whether these oral health inequalities continue as CYP in care progress to become care leavers, but it is likely that this group faces challenges in accessing care. 

Poor oral health is largely preventable and can impact the quality of life, school performance, nutrition, development and a child’s ability to socialise or play [15]. Whilst oral health problems for CYP in care are significant, relevant research is limited. Although a scoping review on the dental health of looked-after children and dental care pathways has recently been published [16], this focuses solely on the UK and does not explore oral health behaviours or oral health promotion interventions in this population. Understanding the oral health profile and behaviours of CYP in care is fundamental to developing recommendations and services to respond to the needs of this population and inform policy. 

This scoping review aimed to determine the global oral health status of CYP in care and their oral health behaviours. It also aimed to outline oral health promotion interventions that have been trialled in this population and their outcomes.

## 2. Materials and Methods

Methods were employed to capture data resulting in two scoping reviews [13]. In this scoping review, three questions were addressed:

(1) What is the prevalence of dental diseases among children and adolescents in care and care leavers?
(2) What are the oral health behaviours of children and adolescents in care/care leavers?
(3) What interventions have been developed for oral health promotion and improvement of care for children and adolescents in care/care leavers?

Due to the variability in the forms of foster care and definitions used internationally, a working definition was determined in accordance with the United Nations guidelines for the Alternative Care of Children (Act 28). This review focused on children and adolescents removed from their biological parents and residing in informal foster care, formal foster care or residential care. Full definitions can be found in the Table S1. A protocol was developed a priori; however, this was not registered on any database. 

### 2.1. Inclusion and Exclusion Criteria

Individual studies were included in the review if they (i) reported on children and adolescents aged 25 years or younger who are currently in formal/informal foster or residential care and care leavers (this age limit was placed in accordance with the UK definition of care leavers); (ii) pertained to oral health profile, behaviours or oral health promotion interventions; (iii) were published in the English language. No restrictions were placed on the date of publication or country. Studies were excluded if they (i) reported on individuals over the age of 25 years; (ii) focused exclusively on dietary data; (iii) were not written in the English language; (iv) were published as only abstracts or posters or were unpublished work.

### 2.2. Search Strategy

A systematic search of the literature related to children and adolescents in care and oral health was conducted by an experienced information specialist (LB) on 3 October 2022, then updated on 8 January 2023 and 2 January 2024. Five electronic databases were searched: Ovid Embase, Ovid MEDLINE, CINAHL (EBSCOhost), SocINDEX (EBSCOhost) and Dentistry and Oral Sciences Source (EBSCOhost). Grey literature sources included the following: Google, EThOS, the Health Foundation, Social Care Online, ClinicalTrials.gov, Fostering Network Voice of the Child in Care, NSPCC and Who Cares Trust, Safeguarding Network, Early Intervention Foundation, Barnardo’s. Search strategies are shown in Appendix A.

The database searches comprised both subject headings and title abstract terms for the concepts of children in foster or residential care and oral health. The terms were derived through scoping searches, discussion with the team and stakeholders. Forward and backward citation searches on the included studies were conducted to supplement the search. No date or country limits were applied. 

### 2.3. Study Records

#### 2.3.1. Data Management/Selection Process

Search results were exported to Endnote (Clarivate Analytics (US) LLC, London, UK) and duplicates removed. Records were then exported to Rayyan [17] whereby two independent reviewers screened all titles, abstracts and full texts against the predetermined eligibility criteria. Any disagreements regarding inclusion were resolved through discussion between the two reviewers. When consensus was not reached, a third reviewer (M.P.) was consulted.

#### 2.3.2. Data Extraction

Data were extracted by the independent reviewers using a predefined data extraction proforma. The form included the following information: author(s) and year of publication; aim and objectives; study design; duration country with associated HDI [18]; sampling method; sample size; participant characteristics (age, gender, ethnicity, socioeconomic status); other participant characteristics (e.g., type of care); setting; description of the intervention (if applicable); data collection tools; main dental health outcomes (oral health profile; oral health behaviours; dental care access/provision; other); results; conclusions; recommendations/gaps in research.

#### 2.3.3. Data Synthesis

A synthesis was informed using the Arksey and O’Malley framework [19]. The six-stage framework was applied to this study as follows: identifying the research question; identifying relevant studies; study selection; charting the data; collating, summarising and reporting the results. Results were summarised to present an overview of the evidence, and quantitative and qualitative analyses were used to describe study characteristics. These analyses allowed major themes to be identified and refined (J.H., H.W. and J.B.) and gaps in the literature to be identified. 

#### 2.3.4. Patient and Public Involvement (PPI)

PPI group and stakeholder representatives were involved in writing and refining the protocol of the review, developing the search strategy and interpreting the results [20]. 

## 3. Results

**Figure 1:**
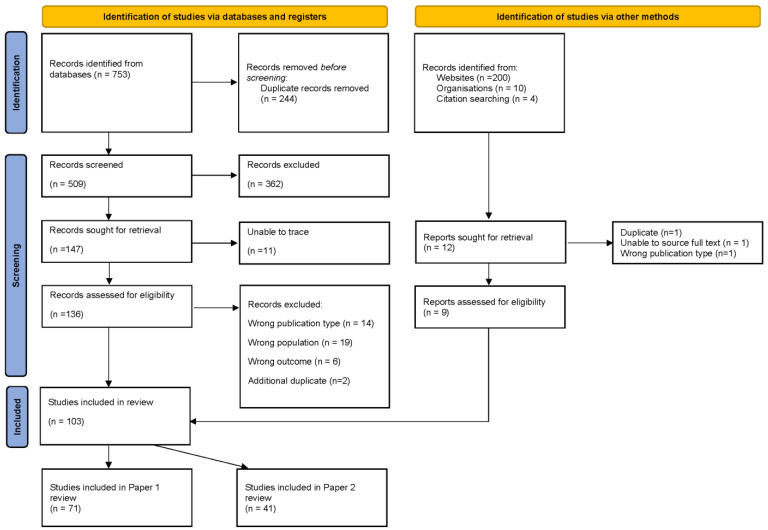
PRISMA flowchart [13].

In total, 71 published papers and grey literature sources were included in this scoping review (Figure 1). The grey literature included a Doctor of Philosophy thesis and commentary, guidance and policy documents. The sources were published between 1994 to 2023 (Figure S1). 

**Figure 2:**
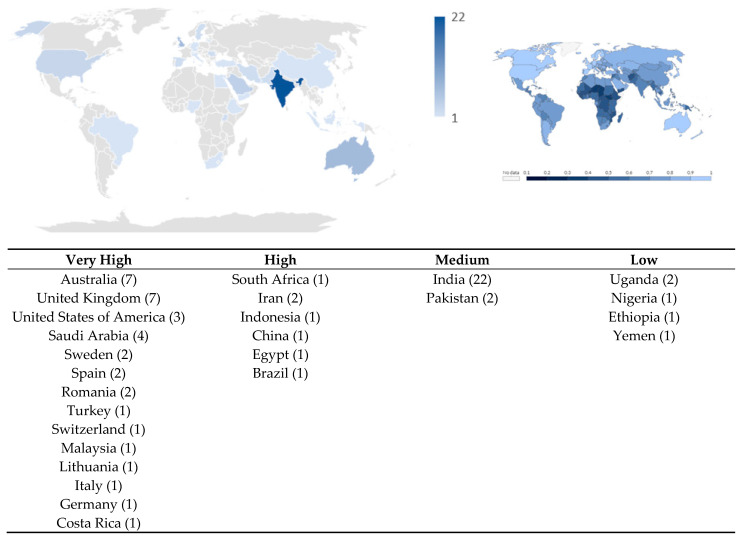
Publication density based on country and HDI category presented alongside a map of the HDI index of countries globally. (n) represents the number of publications published per country.

Thirty-six of the sources were set in very high HDI countries, seven in high, twenty-four in medium and four in low HDI countries (Figure 2). The published study designs included cross-sectional studies, case-control studies, interventional, quasi-experimental, randomised clinical trials and qualitative studies. 

Ages of those involved in the studies ranged from 0 to 19 years. Residency types varied across the studies from orphanages, out-of-home care, foster care, residential care homes, kinship, asylum centres and refugee camps. The number of CYP in care included in the studies varied greatly from 30 [21] to 4696 [22]. When control groups were included, study samples ranged from 30 [21] to 154,774 [22].

### 3.1. What Is the Prevalence of Dental Diseases among Children and Adolescents in Care and Care Leavers?

Studies predominantly detailed the prevalence of dental disease in a single population of CYP in care; however, four of the studies compared this to a comparative population of a similar age who were not residing in care settings [23,24,25,26]. Two of the studies were interventional [27,28], one was a retrospective cohort study [29] and the remaining eight were editorials, health reports, commentaries or service evaluations [30,31,32,33,34,35,36,37]. Half of the studies included were based on CYP residing in very high Human Development Index (HDI) countries (see Table 1). No studies were identified that presented the prevalence of dental disease in care leavers. 

Due to the heterogeneity of the studies in relation to their study design, setting and differences in the methods used to measure dental disease, data have not been pooled together statistically. Instead, a narrative summary was used to synthesise data presenting the overall trends and principal findings. In ten of the studies, the specific oral health needs were not reported; however, “dental problems” were cited as being common in CYP in care [27,31,33,35,36,37,38,39,40,41,42].

#### 3.1.1. Dental Caries

Thirty-eight papers presented data on the decay experience of CYP in care. The prevalence of decay experience was typically measured through DMFT/dmft indices recorded by assessors during site visits. Other methods included using the International Caries Detection and Assessment System (ICDAS) [29,43,44] or recording the signs of untreated dental caries through PUFA, a measure of the clinical consequences of untreated decay [45,46,47]. Most studies reported that among CYP in care there was an increased prevalence of caries [26,48,49,50]; the prevalence varied greatly amongst the studies ranging from 34.8–96.6% [46,51,52,53] and a DMFT/dmft of 1.04–9.01 [24,46]. Dental decay was prevalent in as many as 65% of unaccompanied asylum-seeking children [32]. However, two studies contradicted these findings. Al-Maweri et al. found that the mean dmft score for CYP in care was significantly lower than in the control group [23], and O’Sullivan et al. reported that almost all children under six assessed in a Romanian orphanage (97%) did not have any caries experience [54].

Five of the studies identified the decay component of the DMFT as being responsible for higher scores [46,47,55,56,57], suggesting higher rates of untreated dental caries. This was further supported by Kling et al. who found that 29% of preschool children and half of children aged 7 to 17 had untreated dental decay [58]. This is further supported by three studies that reported PUFA scores [45,46,47]. The prevalence of children with any PUFA signs ranged from 15.9 to 37.7% [46,47], with a mean PUFA score of 1.18 [46]. These studies found that PUFA scores were higher for CYP in care than for their peers [45,46,47]. Rampant early childhood caries were present in 21.8% of children [59]. 

#### 3.1.2. Periodontal Disease

Gingival health was reported in 14 of the studies through the presence of symptoms such as bleeding gingiva or a clinician diagnosis of either gingivitis or periodontitis. Studies indicated that gingivitis was more common in CYP in care rather than in children in the control groups [57,60,61]. The prevalence of gingivitis in this population ranged from 8.8 to 79.49% [37,57,60,62,63,64] and was linked to plaque levels [53,56,65]. Two studies looked at the prevalence of periodontitis. Oliván-Gonzalvo, 2021, found no evidence of periodontitis in a group of unaccompanied immigrant migrants [66], whilst the other study reported that a diagnosis of periodontitis was more prevalent in children in foster care than children in the general population, although this difference was not statistically significant [61]. 

#### 3.1.3. Oral Hygiene

All but two of the studies that looked at oral hygiene [64,67] reported that the oral hygiene of CYP in care was poor [24,28,34,37,53,54,56,60,62,65,68]. Studies indicated that plaque control was worse for CYP in care in comparison to their peers [24,65]. It was suggested that ineffective brushing technique could be a contributing factor, as plaque and calculus levels were high in teenage CYP in care, despite reporting twice daily brushing [34]. Additionally, poor oral health knowledge was associated with poorer oral hygiene [56]. The presence of calculus was also more frequently seen in CYP in care in comparison to their peers [54,60,63,69]. 

#### 3.1.4. Dental Trauma

Six of the papers presented data on dental trauma. The studies indicated that CYP in care had high rates of dental trauma, ranging between 2.1% and 21% [51,66,69,70,71,72]. O’Sullivan et al., 1997, who looked at children residing in an orphanage in Romania, reported 139 fractured incisors in 505 children, all of which were untreated [54]. Alsaif et al. 2022 found a dental trauma prevalence of 27.3% in young people engaged in offending who were living in care houses, which was statistically significantly higher than those in mainstream schools [45]. AlSadhan et al. suggested that whilst children in the general population saw a decrease in dental trauma as they got older, this was the opposite for children in care whose prevalence of dental trauma increased [71].

#### 3.1.5. Occlusion and Orthodontic Treatment Need

Orthodontic treatment need was predominantly measured through the Dental Aesthetic Index (DAI) [45,73,74]. The prevalence of definite malocclusion ranged from 2.9% to 10.3%, severe malocclusion from 2.5 to 6% and very severe malocclusion between 0.6% and 6.1% [45,73,74]. It was indicated that 46% of CYP in care had a definite need for orthodontic treatment, which is a higher proportion than other children their age [34]. Ashok et al.’s paper offered the most detailed insight into the orthodontic treatment need of CYP in care. Furthermore, 1.1% of CYP had either a missing incisor, canine or premolar. The most common malocclusion was class I malocclusion (69.8%), followed by class II div 1 (8.9%) and class III (4.1%), with class II div 2 being the least common (0.5%) [74]. Chernoff et al. did not report on the frequency of malocclusion but instead reported on dental problems of which a component was orthodontic problems [30]. 

Ehlers et al. indicated that whilst the need for orthodontic treatment in CYP in care with ADHD was higher (12%) than in CYP in care without ADHD (4%), the frequency of children having ongoing orthodontic treatment was lower at 6% in comparison to 18% in the control group [62]. 

#### 3.1.6. Temporomandibular Disorders

Two studies looked at the prevalence of temporomandibular disorders (TMD). Sermet Elbay et al. showed that the prevalence of TMD signs and symptoms were significantly higher in CYP in care than in their counterparts [75]. The other study indicated that the prevalence of TMD signs ranged from 27 to 39% in this population [76].

#### 3.1.7. Tooth Surface Loss

Only three studies reported on tooth surface loss [62,69,77]. In all studies, TSL was measured via a clinical examination. In the study conducted by Ehlers et al., teeth were visually evaluated for the presence of TSL: if excessive TSL was present, then these children were classified as being bruxists. The prevalence of bruxism in this study was 56% [62]. Rubin et al. present a more detailed description of TSL in this population, with 15.7% of teeth showing signs of erosion and 32% of children found to have teeth with signs of attrition [69]. Neither of the studies compared the prevalence of TSL in children in the general population. Rodolfo Tavares de Melo et al. looked specifically at CYP residing in shelters in Brazil that house those who are victims of neglect, abandonment or violence. They found that there was no statistical relationship between those who exhibited self-injurious behaviours and tooth surface loss [77].

#### 3.1.8. Soft Tissue Pathologies

Five studies presented findings on the prevalence of soft tissue pathologies and oral medicine conditions in this population [23,49,59,63,77]. Soft tissue disease was significantly higher in CYP in care [48]. Soft tissue pathologies included fissured tongue (24.3%), herpes labialis (7.9%) and traumatic ulcers (2.5%). The prevalence of herpes labialis was found to be significantly higher in CYP in care in comparison to matched controls [23]. This trend was also seen with aphthous ulcers [63]. Blignaut et al. found that oral candidiasis was common among a group of CYP in care who were HIV positive, with some of them developing associated bleeding and ulceration [59]. Rodolfo Tavares de Melo et al. found that linea alba was the most common oral lesion seen in CYP [77].

#### 3.1.9. Oral Health-Related Quality of Life

Of the ten studies that reported on the impact of dental health on Quality of Life, all studies showed a negative impact of poor dental health on QoL [22,25,34,45,59,63,67,72,78,79]. Examples of this included 32% of 12 to 15 year olds reporting that poor oral health impacted their daily lives [34], reports of dental pain [22,59,63,72,79], dissatisfaction with the appearance of their teeth, with only 57–68% of CYP in care reporting that they were happy with their smile [67,78], and poorer social wellbeing than their peers [79]. Both caries experience and malocclusion were significantly associated with lower QoL [45,63,79]. Kamyabi et al. found that of those children who did not have parental care, all experienced functional impairments, 18.5% experienced communication and social challenges and 6.7% reported oral symptoms. All of these parameters were greater in the group of CYP without parental care [25].

### 3.2. What Are the Oral Health Behaviours of Children and Adolescents in Care/Care Leavers?

In total, 23 papers were identified that referenced the oral health behaviours of CYP in care. One of the studies included care leavers [80]. Oral health behaviours were grouped into two themes: (1) oral health self-care behaviours and (2) oral health knowledge and perceptions. 

#### 3.2.1. Oral Health Self-Care Behaviours

The use of a toothbrush varied across studies. One study reported a significant difference in the use of a toothbrush, with CYP residing in orphanages reporting the most common tooth-cleaning tool being their finger compared to non-orphans who had access to a toothbrush [23]. Other studies reported that 64% of CYP in care used a toothbrush [81]. One further study indicated that admittance to an orphanage increased the use of a toothbrush from 72% to 100% [47]. 

The frequency of reported tooth brushing among children in care varied from less than once a day [23,53] to daily [21,25,82,83] or at least twice a day [34,67,83,84]. Factors that influenced the frequency of toothbrushing included age, whereby younger children (7–12 years) were more likely to clean twice a day than those aged 13–17 years, although this finding was not significant [85]. Gender also played a role, with males being less likely to brush twice daily than females [85]. None of the studies directly compared toothbrushing habits across multiple settings of care; however, all the studies that reported a brushing frequency of less than once a day were conducted in orphanages [23,53]. Two studies reported on the duration of toothbrushing; one found that 26% of the children did not brush their teeth for more than one minute per day [21] and identified that only 47% of children who brushed their teeth did so for the recommended duration of two minutes or more [67].

All studies reported on the use of adjunct oral hygiene aids related to CYP living in formal residential care, with one of these studies reporting on the views of caregivers [86]. The use of dental floss varied from 9% [84] to 26% [67]. The use of mouthwash in this population was 23% [67], with 11% reporting using both dental floss and mouthwash. Kanyobi et al. compared those in care to those with parental care and found that of those without parental care only 2% flossed at least once a day compared to 12% of CYP with parental care [28]. Hans et al. reported that 80% of CYP in care regularly rinsed their mouths with water after meals [78]. Several studies cited no evidence of adjunct oral hygiene aids being used [28,47]. 

Two studies cited challenges to accessing oral health products as being a barrier to carrying out oral health self-care behaviours. Challenges stated were a scarcity of resources [86] and the cost of oral hygiene products such as toothbrushes [87]. One of the studies stated that 70% CYP in care replaced their toothbrushes within six months [56]. Saleem et al. and Dumitrache reported that toothbrushes were replaced every three months [67,86].

#### 3.2.2. Oral Health Knowledge and Perceptions

On average, CYP entering care have limited oral health knowledge as they were not taught the skills needed to look after their teeth by their birth parents or taught about the importance of brushing [36,80]. On entering care, CYP not receiving oral hygiene instructions was common [56,88], despite research indicating that being shown how to clean their teeth could result in a 40% decline in decay experience [43]. Oral health-based knowledge remained low even in very high HDI countries [29,62]. Examples of low oral health-based knowledge included 68% of CYP in care believing that consuming sugar was not harmful to dental health, 78% being unaware that plaque deposits can cause decay or gum disease and 65% being unaware of how to control dental plaque [82]. In the same study, 44% of CYP in care did not feel that regular toothbrushing would prevent all dental problems and 15% felt that regular dental visits were not important for maintaining oral hygiene [82].

The oral health perceptions of CYP in care indicated that there was a lack of understanding of the role oral hygiene plays in wider health, with 46% not being aware of the association between oral hygiene and general health [82]. Care leavers, however, spoke of the importance of being taught how to brush properly when entering care, regardless of age [80]. 

This lack of perceived importance of children’s oral hygiene extended to caregivers. One study identified that some caregivers believed CYP coming into their care were too young to experience dental problems and did not view oral hygiene as a matter of health. The oral hygiene practices shared with CYP by caregivers varied greatly depending on regional, cultural, religious and personal differences [86]. For example, in Islamic religious care settings oral hygiene was considered as a virtue and the use of Miswak, a teeth-cleaning stick was common practice. In contrast to this, a study conducted in a very high HDI country, which used a qualitative approach to understand the oral health attitudes of foster carers found that carers were hypervigilant about monitoring oral health practices and were knowledgeable about the causes and consequences of poor oral health [89]. Their perception was that oral health caregiving was an integral part of looking after children’s wellbeing. 

### 3.3. What Interventions Have Been Developed for Oral Health Promotion/Care for Children/Adolescents in Care/Care Leavers?

Eight studies were identified that involved interventions to promote good oral health for CYP in care. Seven of these studies all involved an aspect of oral health education [21,28,85,88,90,91,92], three also included an element of supervised brushing [28,85,90] and two of the studies had an additional component of dental treatment or preventative care [88,92]. 

#### 3.3.1. Oral Health Education

Delivery modes of oral health education varied throughout the studies; audio-visual methods were the most commonly used [21,28,90,91]. In all of the studies, oral health education was delivered directly to the children, and in two of the studies, this was also delivered to caregivers [28,91]. The educational sessions varied in their delivery between group presentations [21] or group presentations accompanied by one-to-one teaching [90,91]. In the Khedekar et al. study, it is unclear whether the education session was delivered to a group or on a one-to-one basis [28]. None of the studies mention the oral health education sessions being delivered virtually. One study involved a single 60 min information session that included a combination of audio-visual content and practical demonstrations of toothbrushing techniques on models. The event increased children’s knowledge on the consequences of not brushing their teeth and perceived likelihood of following the new instructions [21]. A further study compared an intervention group that received audio-visual and individual demonstration of the Bass brushing technique with reinforcement at 14 days follow-up to a control group that received the individual demonstration only. Post-intervention measures at 21 days showed children in the intervention group exhibited significantly higher total oral plaque control and significant reduction in the gingival inflammation (*p* < 0.05) [90]. Where toothbrushing was unsupervised, Shekhar et al. looked at the efficacy of using electric toothbrushes in comparison to manual toothbrushes in this cohort. This was following an audio-visual presentation and then tailored demonstrations for each brush on study models. The study suggested that electric toothbrushes were more effective in comparison to manual brushes at reducing plaque and gingivitis levels [91].

#### 3.3.2. Supervised Toothbrushing

Two of the studies utilised carers to reinforce oral health messaging and supervise toothbrushing [28,90]. Vijayasekaram et al. had caregivers supervise the brushing of both the intervention and control group [90], both groups were given a one-to-one demonstration of the modified Bass technique, and the intervention group was given an additional audio-visual presentation of the technique as well as a reinforcement session after two weeks. Both groups had a reduction in plaque score with the reduction only sustained for the intervention group after two weeks. In addition to oral health education, Khedekar et al. asked carers to supervise and reinforce the toothbrushing technique. This improved oral hygiene and gingival status, but no change was seen in the DMFT score during the two-month follow-up period [28]. Markeviciute et al. compared a group that received educational content to a group that received weekly supervised brushing and practical teaching on brushing techniques delivered by a member of the research team. The group that received the weekly supervised brushing saw a greater improvement in oral hygiene than the group that received only oral health education lectures [85]. 

#### 3.3.3. Dental Treatment or Preventative Care

Fadjeri et al. delivered dental treatment in the form of extractions and fillings using mashed garlic covered with temporary fillings to the intervention group and compared this to a control group that used warm salt mouth rinses. Both groups were given oral health education; those in the intervention group saw a statistically significant decrease in PUFA index after the intervention (1.44 to 0.47 *p* = 0.001) and an increase in nutritional status as measured through body mass index (17.55 to 17.77, *p* = 0.011) [92]. 

Muralidharan et al. designed an intervention with a comprehensive preventative and treatment protocol for a single group with no comparator. The protocol included oral health education, professional oral prophylaxis, weekly mouth rinsing with 0.2% sodium fluoride, biannual topical fluoride application, pit and fissure sealants on all first permanent molars and the provision of any necessary dental treatment including restorations, pulp therapy and extractions. Following the intervention, there was a significant decrease in the mean number of decayed teeth and an increase in the number of filled teeth. This was echoed by a reduction in treatment need for restorations, extractions and pulp therapy [88]. 

## 4. Discussion

### 4.1. Statement of Principle Findings

This scoping review aimed to map and synthesise the current literature surrounding the oral health needs of CYP in care, their oral health behaviours and interventions to improve the oral health of this group. This is the first review of its kind to look at a global perspective of these parameters for CYP in care. Research relating to the oral health of CYP in care has increased steadily from 2011 with a more rapid increase from 2018 onwards. The majority of studies published are from very high HDI countries, followed by medium HDI countries with a lack of evidence representing the oral health needs and behaviours of CYP in care living in high or low HDI countries (Figure S1). In this review, the studies indicated that CYP in care are at risk of poor oral health including dental decay, periodontal disease, dental trauma and soft tissue conditions. As CYP in care got older, their risk of dental trauma increased, whereas this risk decreased for children in the general population [71]. Orthodontic treatment needs were also higher in CYP in care in comparison to their peers in the general population of the same age. A lower oral health related quality of life was also reported for CYP in care with children being unhappy with the appearance of their teeth and suffering from severe dental pain. Both caries experience and malocclusion were linked to a lower QoL [45,63,79]. 

Even though CYP in care have higher oral health needs, they were less likely to carry out oral health self-care practices. Oral health-based knowledge was poor among CYP in care even in high HDI countries and there was a perceived lack of importance regarding oral health. Despite these disparities, there were a limited number of interventions to address the higher needs and oral health risk factors of this group. The type of interventions assessed in this population were predominantly limited to oral health education, none of which used a motivational interviewing approach. A small number of studies explored the impact of supervised brushing, the details relating to the methodology of these supervised brushing schemes were limited and it is unclear whether these were in line with current UK guidance [93]. 

### 4.2. Comparison with Existing Literature 

A recently published scoping review into the dental health and dental care pathways for CYP in care in the UK identified that the oral health needs of CYP in care are greater than among their peers, echoing the findings of our review [16]. However, this review outlined the oral health needs of CYP in care in the UK, whereas the present review provides a worldwide overview suggesting that these disparities are a global phenomenon. The inequalities experienced by CYP in care are similar to other vulnerable CYP populations, for example, those with autism who also experience a high prevalence of dental disease [94]. However, whilst both groups are comparable in terms of their high oral health needs, the challenges these groups face are different. For example, for CYP with autism, a key challenge is providing oral health care for individuals with sensory sensitivities, whereas for CYP in care, it appears that the challenges are more linked to the context of CYP’s living circumstances [95]. Relevant factors in that respect include frequent relocation between care placements [96], variability in who has parental responsibility to consent to dental treatment [14], the instability caused by social workers leaving when CYP in care have formed strong relationships with them [97], poor inter-agency communication and missing routine health surveillance opportunities [98]. 

The findings of this review indicate that the oral health inequalities experienced by CYP in care reflect those seen in other parameters of health such as general, mental health and development [6,7,8]. A longitudinal study conducted in England and Wales suggested that these inequalities extend well into adulthood with those who had been in any type of care reporting poor health even 30 years after leaving care [99]. However, no comparable studies of this nature exist to indicate what the long-term implications of being in care are on oral health. 

### 4.3. Implications for Clinical Practice, Education and Policy

This review provides an evidence-based synthesis that can be used to improve practice, education and policy worldwide in order to meet the unmet oral health needs of CYP in care. It emphasises that CYP in care face similar oral health inequalities globally. Thus, the conclusions drawn from this review can be translated to multiple contexts to inform policy and practice once tailored appropriately for the relevant cultural, social and economic context. Oral health needs should be integrated into routine need assessments and health checks for CYP in care. Clinicians treating and managing the oral health of CYP in care need to have an appreciation that the level of dental disease for care experienced children is likely to be higher than it is for their peers. It is therefore essential that effective treatment and preventative care is delivered for this group. To best facilitate the treatment of CYP in care, clinicians need to be compassionate and understanding of this group that is at risk of social exclusion. CYP in care face multiple disadvantages that can make adopting good oral health practices challenging, for example, living in deprivation might mean they are unable to financially afford toothbrushes, toothpaste or adjunct oral hygiene products. 

Interagency working is likely to support the delivery of dental care by providing continuity as CYP in care move through the care system. Additionally, carers, support workers and social workers are well positioned to be able to deliver oral health advice to CYP in care, ensuring that they can receive the oral health education they may have missed out on earlier in life. Therefore, it is vital that foster carers, residential care practitioners and social workers receive oral health training and provide the appropriate support CYP in care and care leavers require to improve their oral health. These groups could also be supported through training to reinforce oral health messaging such as having an awareness of the wider implications of poor oral health. Training should also be provided to enable them to notice the signs and symptoms of oral disease to signpost CYP in care to dental services. A possible way to improve access could explore linking dental practices with local authorities to provide services for CYP in care and care leavers that are free at the point of access, overcoming the financial barriers to accessing dental care. This care needs to extend to care leavers and be appropriately communicated to the relevant parties so that they are aware that this support exists. 

This scoping review also highlights areas where there is a scarcity of research to inform research priorities. Such areas include understanding the oral health needs and behaviours of care leavers and the long-term impact on the oral health that those being in care experienced. Additionally, currently, CYP in care or care leavers are not identified as a discrete group in national dental health surveys. Changing policy to adapt national surveys to capture data on the dental needs of CYP in care would ensure that the needs of this group are better understood, and this has been recommended in previously published literature [16]. 

### 4.4. Strengths and Limitations of the Scoping Review 

A strength of this scoping review is that it is the first paper published that provides a global perspective on the oral health status of CYP in care and their oral health behaviours. Additionally, it is the first paper that the authors are aware of that synthesises interventions that have been utilised in this population to improve their oral health. However, the global nature of the papers included in this review makes it challenging to interpret the results as the contexts in which the studies were conducted are vastly different and relate to different CYP in care or care leave populations. Due to this, care needs to be taken when applying the results of this review to different CYP in care and care leaver populations. A scoping review was selected as the methodology of choice as it can be used to synthesise the nature and extent of the research evidence linked to the oral health of CYP in care [100]. This scoping review used rigorous and transparent methods throughout the entire process, with more than one reviewer involved in study selection to reduce selection bias. A further strength of this scoping review methodology was the inclusion of grey literature sources. The inclusion of grey literature reduces publication bias and provides a more comprehensive overview of the current evidence base [101]. 

A key limitation of this scoping review is that it relies on the methods deployed in the studies for data collection. Conducting fieldwork style examinations in sites such as orphanages is necessary for practicality purposes especially in developing countries where thorough clinical examinations may not be feasible. When assessing the oral health status of CYP in care, many studies were conducted as fieldwork with researchers going out into residential settings to measure the presence of dental disease. This technique reduces the reliability of the results as it is likely to underestimate the level of disease in comparison to diagnoses made in a dental clinic with adequate lighting, assessment tools and radiographs. However, this fieldwork at least provides some insight into the oral health status of CYP in care. One of the limitations of this study is the inclusion criteria, as only sources published in English were included. A truly representative global perspective may not be presented as studies from non-English speaking countries may have been excluded. Additionally, abstracts and conference presentations were excluded, introducing a degree of publication bias. 

Another key limitation of this scoping review is that in assessing the oral health behaviours of CYP in care, several risk factors such as smoking or alcohol intake were not included. Dietary data were included if they were in a dental context. It is possible that some studies looking purely at diet and sugar intake outside of a dental context have been included. These factors such as a cariogenic diet are known to play a role in the development of oral disease [102] and could be targeted in oral health promotion strategies to decrease the prevalence of oral disease in this population. Additionally, this scoping review did not appraise the quality of the studies included when summarising the results; therefore, it is not possible to determine gaps in the current evidence base due to low-quality research, and caution needs to be taken when applying the conclusions drawn to make recommendations or change policy. 

### 4.5. Unanswered Questions and Future Research Directions

There is a significant gap in the literature regarding the oral health of care leavers. As young people transition out of care, the support available to them reduces and they become particularly vulnerable. With care leavers being left to navigate health systems independently, it is possible that they may struggle to access dental care and continue to experience poor oral health [103]. Understanding the oral health needs and behaviours of care leavers will allow for the development of specific recommendations for this group as it is likely that their needs and behaviours will differ from CYP in care. Furthermore, CYP in care are at risk of entering other vulnerable populations in adulthood such as those experiencing homelessness or those in contact with the criminal justice system. Both populations face further challenges to achieving good oral health [104,105]. 

Whilst oral health education and the importance of preventative strategies have been outlined in the studies included in this review, currently, there is a limited evidence base to support oral health promotion strategies for this group. Further evidence is needed to determine what the best approach might be to feasibly deliver oral health interventions to CYP in care and care leavers. Having a better understanding of this will allow the integration of oral health promotion into current care systems to improve the oral health outcomes of this group. There was also a paucity of evidence from low and high HDI countries, and a better understanding of the oral health experiences of CYP in care in these countries is fundamental to adapting tailored approaches for these countries. 

It is unlikely that a blanket approach will be feasible when addressing the high prevalence of poor oral health in CYP in care. Further mixed-method research is needed to better understand the nuances within the different forms of care and between countries of varying HDIs to inform the development of future interventions. Research should take a participatory approach involving CYP in care, care leavers and care experienced young people to set research priorities, aid recruitment and improve impact.

## 5. Conclusions

This scoping review presents international evidence indicating that CYP in care face oral health inequalities, with CYP in care experiencing a higher prevalence of poor oral health and limited oral health behaviours. There this a lack of oral health interventions for this population to address these issues. There is a need for further research to explore the factors that influence oral health behaviours and to understand the oral health needs of care leavers. 

## Figures and Tables

**Table 1:** Number of studies categorised by Human Development Index (HDI) and oral health outcomes, behaviours and interventions. Some studies provide an insight into multiple questions and several parameters within the research questions.

	Total Number of Studies			
Oral Health Outcomes (*n* = 59)	Low *n* = 5	Medium *n* = 19	High *n* = 4	Very High *n* = 31
Dental caries/PUFA	6	14	3	18
Periodontal disease	1	5	1	7
Oral hygiene	2	4	2	8
Dental trauma	1	1	0	5
Occlusion and orthodontic treatment need	1	2	0	5
Tooth surface loss	2	0	0	1
Temporomandibular disorders	0	1	0	1
Oral medicine conditions	2	2	1	0
Oral health related quality of life	0	3	2	5
General dental health	0	0	0	10
**Oral Health Behaviours (*n* = 23)**	**Low *n* = 2**	**Medium *n* = 9**	**High *n* =** 1	**Very High *n* = 11**
Oral health self-care	2	7	1	6
Oral health knowledge and perceptions	0	5	0	5
**Oral Health Interventions (*n* = 8)**	**Low *n* = 0**	**Medium *n* = 4**	**High *n* = 1**	**Very High *n* = 3**
Oral health education	0	4	0	2
Dental treatment or preventative care	0	1	1	0
Supervised toothbrushing	0	2	0	0

## Data Availability

The data that supports the findings of this study are available in the Supplementary Material of this article in the data extraction table.

## Supplementary Materials

The following supporting information can be downloaded at: https://www.mdpi.com/article/10.3390/dj12020038/s1, Table S1: Definitions of types of care; Figure S1: Number of publications by year and HDI index category; Table S2: Data extraction table.

## Appendix A. Search Strategy

**Table A1:** Search Strategy.

1	(child* or youth* or adolescen* or teen* or young people).tw.
2	care leaver*.tw.
3	leaving care.tw.
4	(transit* adj3 (care or services)).tw.
5	child* in care.tw.
6	looked after child* .tw.
7	accommodated child.tw.
8	(“out of home care” or “out of home placement”).tw.
9	kinship care.tw.
10	((adoption or adopted) adj3 child*).tw.
11	adopted child/
12	(custod* adj3 care).tw.
13	orphan*tw.
14	(placement adj3 care).tw.
15	public care.tw.
16	(foster adj1 (care* or home* or family or parent*)).tw.
17	(institutional* adj3 (care or home*)).tw.
18	(group adj1 home*).tw.
19	(residential adj3 (care or home* or facilit*)).tw.
20	(welfare adj3 (care or system*)).tw.
21	statutory care.tw.
22	(care ajd3 local authority).tw.
23	care order*tw.
24	(substitute adj1 (care or famil*)).tw.
25	special guardian*.tw.
26	Kafalah.tw.
27	(unaccompanied adj3 asylum).tw.
28	(unaccompanied adj3 refugee*).tw.
29	(“Children Act 1989” or “Children Northern Ireland Order 1995” or “Children Scotland Act 1995”).tw.
30	or/5–29
31	dental health/
32	dental procedure/
33	exp tooth disease/
34	exp dentist/
35	(oral adj3 (health* or hygiene or care)).ab,kw,ti.
36	dental.ab,kw,ti.
37	((tooth adj3 (health* or hygiene or care or brush* or floss*)) or toothbrush*).ab,kw,ti.
38	(teeth adj3 (health* or hygiene or care or brush* or floss*)).ab,kw,ti.
39	dentist*.ab,kw,ti.
40	or/31–39
41	(1 or 2 or 3) and 30 and 40

* Search strategy adapted for each database < 1974 to 2 January 2024 >.
