# Supplementary material for: A Scoping Review of the Oral Health Status, Oral Health Behaviours and Interventions to Improve the Oral Health of Children and Young People in Care and Care Leavers
Source: Dent J (Basel). 2024 Feb 9;12(2):38. doi: 10.3390/dj12020038 (PMC10887692; doi:10.3390/dj12020038)

Supplemental data:

S1:

Formal foster care	"Situations where children are placed by a competent authority for the purpose of alternative care in the domestic environment of a family other than the children's own family that has been selected, qualified, approved and supervised for providing such care"
Informal foster care	"Any private arrangement provided in a family environment whereby the child is looked after by relatives or friends...or by others in their individual capacity, at the initiative of the child, his/her parents or other person without this arrangement having been ordered by an administrative or judicial authority or a duly accredited body"
Residential care	"Care provided in any non-family-based group setting, such as places of safety for emergency care, transit centres in emergency situations, and all other short- and long-term residential care facilities, including group homes"

Definitions of types of care as defined by The Guidelines for the Alternative Care of Children (Art.28)

S2: A chart showing the number of publications by year and location (very high, high, medium and low HDI).

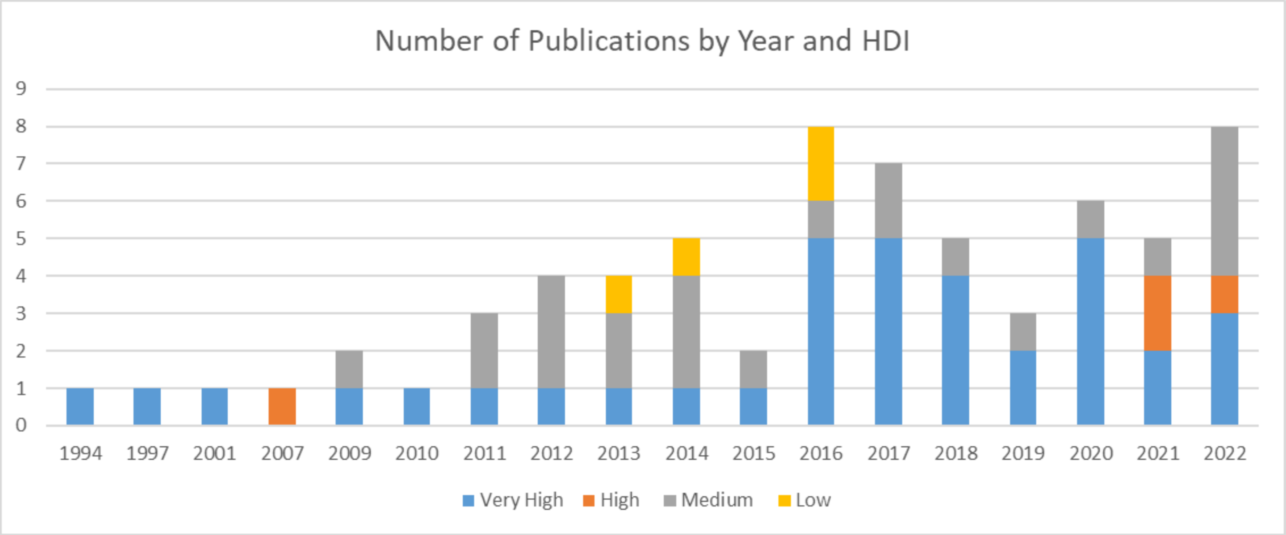

Supplement: Supplementary file 1 [file dentistry-12-00038-s001.zip › CLA Oral Health Status Supplemental data.pdf]
